# Consensus guidelines for the management of HR-positive HER2/neu negative early breast cancer in India, SAARC region and other LMIC by DELPHI survey method

**DOI:** 10.1186/s12885-023-11121-9

**Published:** 2023-07-31

**Authors:** Purvish Parikh, Govind Babu, Randeep Singh, Vamshi Krishna, Amit Bhatt, Indu Bansal, Senthil Rajappa, Tarini Prasad Sahoo, Shyam Aggarwal, Ajay Bapna, Ghanshyam Biswas, SP Somashekhar, Jyoti Bajpai, Vashishtha Maniar, Sharad Desai, T Raja, Goura Kishor Rath

**Affiliations:** 1 Dept of Clinical Hematology, Mahatma Gandhi Medical College Hospital, Jaipur, 302023 India; 2 HCG Cancer Hospital, Bengaluru, 560027 India; 3 Narayana Super speciality Hospital, Gurugram, 122002 India; 4 Asian Institute of Gastroenterology, Hyderabad, 500082 India; 5 Avinash Cancer Clinic, Pune, 411004 India; 6 Basavaratakam Indo American Cancer Hospital & Research Institute, Hyderabad, 500034 India; 7 Silverline Hospital, Bhopal, 462001 India; 8 Sir Gangaram Hospital, New Delhi, 110060 India; 9 Bhagwan Mahaveer Cancer Hospital and Research Centre, Jaipur, 302017 India; 10 Sparsh Hospital & Critical Care, Bhubaneswar, 751007 India; 11 Manipal Comprehensive Cancer Center, Manipal Hospital, Bengaluru, 560017 India; 12 Tata Memorial Hospital, Mumbai, 400012 India; 13 Mumbai Oncocare Center, Mumbai, 400086 India; 14 Mahatma Gandhi Cancer Hospital, Miraj, 416410 India; 15 Apollo Speciality Cancer Hospital, Chennai, 600035 India; 16 DR. B.R.A. Institute Rotary Cancer Hospital, Delhi, 110029 India

**Keywords:** Personalized therapy, Precision oncology, Low- and middle-income countries, Avoiding toxicity, Avoiding financial distress, Saving lives, Practical recommendations, Prognostic tests, Multigene tests

## Abstract

**Background:**

Precise prognostication is the key to optimum and effective treatment planning for early-stage hormone receptor (HR) positive, HER2/neu negative breast cancer patients. Differences in the breast cancer incidence and tumor anatomical features at diagnosis, pharmacogenomics data between Western and Indian women along with the vast diversity in the economic status and differences in insurance policies of these regions; suggest recommendations put forward for Western women might not be applicable to Indian/Asian women. Opinions from oncologists through a voting survey on various prognostic factors/tools to be considered for planning adjuvant therapy are consolidated in this report for the benefit of oncologists of the sub-continent, SAARC and Asia’s LMIC (low and middle-income countries).

**Methods:**

A three-phase DELPHI survey was conducted to collect opinions on prognostic factors considered for planning adjuvant therapy in early-stage HR+/HER2/neu negative breast cancer patients. A panel of 25 oncologists with expertise in breast cancer participated in the survey conducted in 2021. The experts provided opinions as ‘agree’ or disagree’ or ‘not sure’ in phases-1 and 2 which were conducted virtually; in the final phase-3, all the panel experts met in person and concluded the survey.

**Results:**

Opinions on 41 statements related to prognostic factors/tools and their implications in planning adjuvant endocrine/chemotherapy were collected. All the statements were supported by the latest data from the clinical trials (prospective/retrospective). The statements with opinions of consensus less than 66% were disseminated in phase-2, and later in phase-3 with supporting literature. In phase-3, all the opinions from panelists were consolidated and guidelines were framed.

**Conclusions:**

This consensus guideline will assist oncologists of India, SAARC and LMIC countries in informed clinical decision-making on adjuvant treatment in early HR+/HER2/neu negative breast cancer patients.

## Introduction

Breast cancer continues to be public health focus in many parts of the world, including the SAARC region and India [1, 2]. Age-adjusted incidence in India is 25.8 per 100,000 women and mortality is 12.7 per 100,000 women, this is increasing since 1982 in all our population-based cancer registries (PBCRs; up to 2.84%); by 2020, it was projected to have as many as 17,97,900 cases [3]. The median age at diagnosis is 44.6 years with a peak between 40 and 50 years [4]. Real-world Indian data showed that hormone receptor (HR) positive and HER2 negative constitute 50–60% of all breast cancer (BC) patients [5].

To improve their outcome, especially with respect to cure, it is crucial to identify high-risk individuals requiring a more aggressive approach as well as separate out low-risk patients to prevent them from getting unnecessary and non-beneficial treatment [6–9]. Because of significant differences in the natural history, disease biology, as well as pharmacogenomics; treatment of Asian patients based on Western guidelines and/or recommendations that lack validation data on Asian patients and more specifically on Indian patients need caution. Such an approach would either result in overtreatment (and associated toxicity–medical, financial, and societal issues) or in undertreatment (reducing cure rates) [8, 9].

In the current manuscript, we conducted a survey by the DELPHI method to collect opinions on various prognostic factors/tools used to estimate the cancer recurrence risk and thereby plan therapy in HR+, HER2/neu negative BC patients; that could be used pragmatically by oncologists of India, SAARC and LMIC countries in day-to-day clinical practice.

## Materials and methods

The DELPHI survey model was conducted in three phases over 12 months. During the first phase, a Steering Committee including 4 experts in breast cancer (BC) was asked to define relevant statements on various topics related to prognostication and therapy in the Indian scenario. After an advisory board meeting, of the 68 statements, the Steering Committee identified 41 preliminary relevant recommendation statements on prognostic factors/tests and adjuvant endocrine treatment in ER-positive early breast cancer (EBC), based on available published data. In the second phase, an expert committee was put together. It consisted of 25 oncologists (medical oncologists-21, surgical-2, radiation-2) specifically dealing with the day-to-day management of breast cancer. They represented several academic organizations, government and private hospitals and societies across India and have an experience in managing large volumes of breast cancer patients. In the second phase, we conducted a voting survey consisting of 3 rounds. In the first round, a web-based survey was carried out under the aegis of the Integrated Academic Society of Clinical Oncology (IASCO). The panelists were asked to express anonymously their level of agreement with each statement, using a three-point Likert scale (where 1 = completely agree; 2 = not sure; 3 = completely disagree) [10]. The panelists were guided by published evidence as well as intricate analysis of practical experience fromthe real-world patient management by national and international experts on all the statements [11]. Our panel experts were provided with previous SAARC publications, including the results of an online poll of oncologists (involving medical oncologists, radiation oncologists, surgical oncologists, molecular oncologists, and policymakers) [12–14]. A consensus was deemed as achieved if “answer 1” exceeded 66%, as described in previous studies conducted with this method [15–17]. In cases where consensus was not achieved, voting process was repeated after the experts were provided additional publications/data. This virtual voting survey was conducted twice, and the last round of voting involved an in-person meeting, held in Bengaluru on 12th Dec 2021. In the final third round, in the in-person meeting, panel experts finalized these consensus guidelines statements for the benefit of community oncologists, so that they would have ready-to-use practical recommendations for India and the SAARC regions. We believe that these guidelines are also applicable to other LMIC [8, 11, 12].

## Results

Of the 41 statements, 30 statements achieved the threshold for positive consensus in the first round. The remaining 11 statements were circulated in the second round with supportive literature from various clinical trials. An additional statement on the treatment of luminal sub-types was formed in the final and in-person rounds. In the end, 35 statements obtained positive agreement. These statements are clustered under various categories of prognostic and predictive factors, online prognostic tools, multigene tests for prognostication and the use of hormonal therapy (Table 1). The recommendation statements that obtained consensus in rounds 1 and 2, along with other recommendations on surgery and radiotherapy are represented in Table 2.

**Table 1:** Delphi survey results

Sl. No.	Category	parameter	Agree	Disagree	Not Sure
1	Which of the following factors are important to assess the risk of recurrence?	Age of patient	88%	12%	0%
2		Tumor Size	96%	4%	0%
3		Nodal Status	100%	0%	0%
4		Histopathological grade of tumor	88%	12%	0%
5		ER expression levels	88%	12%	0%
6		Ki67 level	88%	12%	0%
7		Gene expression profiling	88%	0%	12%
8	On what factors is the decision for use of chemotherapy dependent?	Age of patient	96%	4%	0%
9		Tumor Size	88%	12%	0%
10		Nodal Status	100%	0%	0%
11		Histopathological grade of tumor	76%	16%	8%
12		ER expression levels	84%	16%	0%
13		Ki67 level	92%	4%	4%
14		Gene expression profiling	92%	0%	8%
15	How useful are the online predictive tools in making decisions on chemotherapy use?	NPI	44%	20%	36%
16		IHC4	56%	20%	24%
17		PREDICT	64%	24%	12%
18	Choice of Multi-marker prognostic tests for deciding on prescribing chemotherapy?	Oncotype DX	80%	16%	4%
19		CanAssist Breast	84%	12%	4%
20		MammaPrint	28%	4%	68%
21		Prosigna	20%	72%	8%
22		EndoPredict	28%	64%	8%
23	Clinical utility of multi-marker prognostic tests	Used routinely	88%	12%	0%
24		When facing a clinical dilemma	84%	8%	8%
25		Based on patient affordability	92%	4%	4%
26		Western tests because they are part of international treatment guidelines	92%	8%	0%
27		Western tests are not ideal / not validated in Indian patients	92%	8%	0%
28		TAILORx study had few Indian/ Asian patients and hence Oncotype DX is not applicable to Asian patients	88%	8%	4%
29	Differences in breast cancer incidence between Asian (including Indian) versus Western women	Asian (including Indian) and Western women with EBC to be treated differently because the biology of the disease is different	76%	24%	0%
30		Are Asian (including Indian) women diagnosed at an earlier age / during premenopausal status?	76%	24%	0%
31		Do Asian (including Indian) women diagnosed at an earlier age (less than 40 years) have more aggressive disease?	84%	16%	0%
32		Do Asian (including Indian) women below age of 45 years have high expression of proliferation genes and genes involved in endocrine resistance?	80%	12%	8%
33		Did the SEER Oncotype DX data show that ethnic background influences breast cancer specific mortality – specifically that black women had higher mortality compared to white women of same type, grade and stage of breast cancer?	88%	4%	8%
34	CanAssist Breast as a prognostic test	Does it predict risk of recurrence based on tumor biology?	84%	8%	8%
35		Does it predict risk of recurrence across ethnic backgrounds and diverse geographies?	76%	12%	12%
36		Is it affordable in LMIC?	80%	12%	8%
37	Use of Hormonal Therapy	Does its use for more than 5 years in postmenopausal women reduce the risk of recurrence as well as the risk of contralateral breast cancer?	92%	4%	4%
38		Is recurrence after 5 years of adjuvant endocrine therapy associated with patients identified as a high-risk group with multi-marker prognostic tests?	80%	20%	0%
39	Use of Hormonal Therapy in male breast cancer patients	Should it also be used for male patients?	100%	0%	0%
40		For male patients, is the preferred choice with Tamoxifen?	96%	4%	0%
41		For male patients, should tamoxifen be combined with GnRHa because it further reduces estradiol levels?	76%	16%	8%

**Table 2:** Practical Consensus Guidelines for optimizing the benefit of chemotherapy in the management of HR positive HER2/neu negative early breast cancer

Sr No	Consensus Guidelines statements
1	Patients with HR-positive HER2/neu negative early breast cancer should be treated with curative intent, unless contraindicated.
2	Primary tumor and regional lymph node assessment are key to optimizing therapy in this potentially curative group of EBC.
3	Surgery is the primary treatment for all patients with HR positive HER/neu negative EBC. In most instances, BCS is the preferred treatment that should be offered. Patients may choose to undergo BCS or MRM.
4	Radiation Therapy is required for all such patients undergoing BCS and selected patients undergoing MRM.
5	Appropriate evaluation is recommended to identify patients requiring neoadjuvant/adjuvant systemic cancer-directed therapy.
6	Patients suspected to have hereditary breast cancer should be evaluated with appropriate testing and counseling.
7	Clinical features alone are not sufficiently robust in separating patients into the low and high-risk categories.
8	Patient features important for predicting risk of recurrence include (in descending order of importance) nodal status; tumor size; (and of equal importance) age of patient, histopathological grade of tumor, ER expression levels, Ki67 level and gene expression profiling results.
9	Features important for making a decision on whether to use chemotherapy or not include (in descending order of importance) nodal status; age of the patient; Ki67 levels and gene expression profiling (of equal importance); tumor size; ER expression level; and histopathological grade of tumor.
10	Online predictive tools cannot be relied on and are not to be used in patient decision making.
11	CanAssist Breast and Oncotype DX are the recommended multi-marker prognostic tests that have substantial documented evidence.
12	Multi-marker prognostic tests should be used routinely if appropriate and if patients can afford them; especially when facing a clinical dilemma.
13	Western guidelines advocate the use of multi-marker risk assessment tools for patients with early breast cancer, based on validation predominantly in the Caucasian population.
14	When tests change their cutoff values and/or have different cutoff values for different age groups (like Oncotype DX), their reliability becomes questionable. This is especially important in Indian patients where a significant proportion (about 50%) are diagnosed in the premenopausal stage (early age).
15	Asian/Indian patients have a biologically different disease which is more aggressive in younger patients and can have higher expression of poor prognostic genes.
16	Regulatory authorities in India, in their breast cancer treatment guidelines, have specifically stated that such tests should not be used in clinical practice unless validated amongst Indian patients.
17	The only currently available predictive test for HR positive HER/neu2 negative EBC that has been validated in Indian patients is CanAssist Breast.
18	CanAssist Breast is also a predictive test that predicts the risk of recurrence; is applicable across ethnic backgrounds and geographies; and is affordable in LMIC.
19	Hormonal therapy with Tamoxifen should be used in male patients.
20	The use of hormonal therapy for more than 5 years reduces the risk of recurrence as well as contralateral breast cancer.
21	Recurrence after 5 years of endocrine therapy occurs in patients who have been identified as having high risk by multi-marker prognostic testing.
22	If an HR positive HER2/neu negative EBC patient demonstrates conflicting risk features (clinical low risk features with biomarker high-risk score OR clinical high-risk features. with biomarker low-risk score [Ex: CanAssist Breast score ≤ 15.5]), the biomarker risk score is more reliable. Informed discussions are recommended with patients before finalizing overall treatment plan to optimize the chance of potential cure in such patients.
23	The use of these practical consensus guidelines will assist real-world patient treatment decision making by avoiding the cost/ toxicity of chemotherapy in patients unlikely to benefit from it. It will also ensure that patients with a high risk of recurrence are correctly selected to receive chemotherapy as part of their potentially curative treatment plan.
24	These practical recommendations are applicable even during the COVID-19 pandemic since patients with HR positive HER2/neu negative early breast cancer are treated with curative intent.

### Diagnostic workup

Although the survey did not include any questions on diagnostic workup, the panelists discussed assessments to be made based on various parameters for each patient during the disease diagnosis. Similarly, opinions on surgery and radiotherapy were consolidated. The steps to be followed for each patient vary on case-to-case basis and the recommendations framed in this survey are in line with guidelines provided by international committees [18]. The diagnostic workup should follow the recommendations mentioned in Table 3. Primary tumor and regional lymph node assessments are key to optimizing therapy in this potentially curative group of EBC. Not all the features are necessary for each patient. Systemic staging of asymptomatic patients is not warranted as routine practice [19]. Further tests are required only when clinically indicated. The bone scan is to be performed in patients with pain in the bone and with elevated alkaline phosphatase if clinically the disease is of stage II [20–22]. Bone mineral density test is recommended in post-menopausal women who would be treated with aromatase inhibitors for more than 5 years, to avoid the risk of fractures in these women. Abdominal with or without pelvic CT or MRI to be done in cases of abnormal LFT, abnormal physical examination of abdomen/pelvis. Hereditary cancer assessment requires appropriate utilization. The panel experts recommended genetic counselling in women diagnosed with high-risk disease.

**Table 3:** Diagnostic workup for HR + ve HER2/neu –ve Early Breast Cancer

Sl. No	Health parameter assessed	Tests required to be done
1	General health assessment	History and menopausal status
		Physical Examination
		CBC (Hb, Total WBC count, % of neutrophils, platelet count)
		Liver function test and alkaline phosphatase as appropriate
		Renal Profile as appropriate
		Cardiac Profile as appropriate
		Bone mineral density test
2	Hereditary cancer assessment	When appropriate, testing for BRCA and other hereditary cancer genes (age less than 50 years, family history of breast cancer, bilateral breast cancer, etc.)
3	Primary tumor assessment	Mammography and/or Breast sonography
		Breast MRI only in selected cases
		Core Biopsy pathology (histology, ER, PR, HER2/neu, Ki67)
		Biomarker profiling validated in the concerned ethnic population
4	Regional lymph node assessment	Sonography of the axilla
		Sonography guided biopsy only in selected cases
5	Metastasis assessment	Additional imaging Tests for assessing the presence of distant metastasis may be done only in selected cases (if high tumor burden, aggressive biology or symptoms indicative of metastasis are present)

### Prognostic factors deciding the risk of recurrence and use of chemotherapy in HR + ve, HER/neu-ve EBC

The expert committee identified the following factors that are important to assess the risk of recurrence in patients with HR + ve, HER2/neu–ve EBC (Table 1). All the committee members considered nodal status as the most important prognostic factor that also predicts chemotherapy benefit. A total of 96% also considered tumor size to be correlating with the risk of recurrence. For the remaining five features (age, histopathological grade, ER expression, Ki67 levels and gene expression profiling) 88% of experts considered them crucial for predicting the risk of recurrence. The experts opined that tumor size, tumor grade, age of the patient at the time of diagnosis, levels of ER, Ki-67 were more or less of similar importance and correlated with chemotherapy benefit (at least 72% of panelists agreed). Higher clinical risk like big tumors, higher nodal involvement, and moderate and high-grade disease was associated with worst survival and higher recurrence rates [23, 24]. Data from clinical trials have shown that women under 50 years derive a survival benefit of 7–11% with chemotherapy while the benefit of 2–3% in patients aged between 50 and 69 [25]. Besides these factors, the role of proliferation markers, ER and Ki-67 in making systemic adjuvant therapy decisions was discussed. More than 80% of panelists voted for the prognostic role of ER and Ki-67. The threshold for ER positivity has undergone revision from 10 to 1%, with data suggesting a limited endocrine therapy benefit in patients with ER lower than 10% [26]. Nonetheless, panelist agreed to 1% of ER as being positive honouring ASCO and CAP recommendations [27]. However, PR status failed to achieve a positive consensus threshold in both rounds 1 and 2 and hence was excluded from the table. In view of the subjective nature of Ki-67 staining and grading, 80% of panelists expressed their opinion that expression levels of Ki-67 of 14% and above are considered high-risk. The latest recommendations from the International Ki-67 in Breast Cancer working group opined that Ki-67 above 30% could be considered high-risk and for patients with Ki-67 between > 5-<30% advice from a prognostic test should be considered for deciding on chemotherapy use [28]. The panelists discussed the importance of gene expression profiles for decision-making on the use of chemotherapy at length. 88% of the panelists voted for the use of prognostic tests that assess the risk of recurrence based on the expression of genes that provide significant information on cancer progression which clinical parameters and proliferation indices might miss out [29]. Hence the consensus guidelines statement is that all seven features are important for assessing the aggressiveness of the disease thereby predicting the risk of recurrence and to be considered for making decisions on whether to use chemotherapy or not (Table 2).

### Utility of online prognostic tools for taking decisions on chemotherapy use

The expert committee evaluated three online predictive tools – NPI, IHC4 and PREDICT. The opinion on their utility was variable (Table 1). NPI uses a simple equation that estimates overall survival based on clinical parameters while PREDICT along with clinical parameters uses information on age, menopausal status, ER and Ki-67 for making survival estimates post-surgery for various adjuvant treatment regimens [30, 31]. Contrary to these two tools, IHC4 is purely based on information derived from immunohistochemistry of ER, PR, Ki-67 and HER2 without considering the clinical parameters [32]. Even after extensive discussion and re-review of updated literature, there was no confidence in the value of such predictive tools. Although these online tools provide reliable prognostic information in some cohorts, they have been shown to overestimate or underestimate survival in patients of certain age groups. NPI is sub-optimal in predicting prognosis in patients < 40 years and underestimated overall survival in patients aged between 55 and 60 years [33]. In a study on a cohort of 600 patients, aged below 40 years PREDICT overestimated chemotherapy benefits and 10-year mortality by 8% [34, 35]. Moreover, NPI and IHC4 have ambiguous intermediate-risk zones, failing to provide a definite treatment option to these patients. The panelists highly opined cancer recurrence and progression are driven by key biological markers, which manifest in upstaging of tumor anatomical features by transforming clinical low risk into high risk. AJCC revised the staging definition from anatomic staging to a prognostic staging system with the incorporation of biomarkers in its 8th edition [36]. As a result, a recent study conducted on 4729 patients with T1-T2N1mi disease with 5-year follow-up found that approximately 84.4% of patients were downstaged and 3.7% of patients were upstaged and reported that 8th AJCC system predicted better breast cancer-specific survival compared to 7th AJCC staging system [37]. Hence the consensus guidelines statement is that these online predictive tools that primarily use clinical parameters to predict survival benefits, are not to be used in patient decision-making (Table 2).

### Use of multi-marker prognostic test in clinical practice

The expert committee spent substantial time discussing the role of multi-marker prognostic tests. Five tests were considered for the discussion in this survey (Table 1). A total of 84% of experts were in favour of CanAssist Breast and 80% were also in the favour of Oncotype DX. The usage of the other three tests ranged between 20% and 28% of the experts. Oncotype DX and MammaPrint are the first-generation prognostic signatures. Oncotype DX is a 21 gene signature that has been developed in the NSABP-14 cohort and validated in NSABP-20, TransATAC and SWOG8814 cohorts. Other than these, Oncotype DX has been validated in large clinical trials in a prospective manner in patients with node-negative (TAILORx trial) and node-positive patients (RxPONDER). MammaPrint is a 70 gene signature validated in prospective trials, RASTER and MINDACT. Prosigna is a 50-gene signature initially developed to distinguish luminal subtypes. This test is validated only in post-menopausal women with different risk zones for node-negative (3 zones) and node-positive patients (2 zones). EndoPredict is a 12-gene signature that later along with clinical parameters (node and tumor size) was called EPClin. This test has been validated retrospectively on ABCSG6 and 8 cohorts [29]. While all the above mentioned tools use logistic regression, CanAssist Breast is an IHC based tool that uses an Artificial Intelligence-based Machine (SVM) algorithm for the prediction of recurrence risk with inputs from immunohistochemistry information of 5 critical biomarkers of cancer progression and recurrence pathways along with 3 clinical parameters [38]. CanAssist Breast has been validated in multiple cohorts from Southeast Asia, the USA and Europe [39–41] and in a DUTCH sub-cohort of TEAM trial- a prospective randomized trial [42]. The consensus guidelines state that CanAssist Breast and Oncotype DX are the recommended multi-marker prognostic tests that are the preferred choice (Table 2).

Other more recent prognostic tools which are not considered in this survey are DigiStain and a test developed in Korea named GenesWell BCT [43, 44]. Both these tests are relatively new in SAARC countries; DigiStain is available for patients in select regions and the Korean test is undergoing additional validation studies to be available for patients, we can perhaps include them in the survey in the next updating of the guidelines.

### Scenarios where multi-marker prognostic tests are used in clinical practice

The appropriate clinical utility of multi-marker prognostic tests was also discussed. A total of 92% of the experts agreed that their use is based on patient affordability and on inclusion in international treatment guidelines (Table 1). An identical number (92%) also agreed that Western tests have not been validated in Indian patients; also 88% said that the TAILORx trial that assessed Oncotype DX performance in node-negative patients included a few Indian/Asian patients and hence is not applicable to this ethnic/geographical group [45]. While 88% used multi-marker prognostic tests routinely and 84% said they recommended use when facing a clinical dilemma in treatment decision- making. The consensus guidelines statement is that multi-marker prognostic tests should be used routinely based on patient affordability and especially when facing a clinical dilemma. Even if Western tests are included in international guidelines they should not be used blindly since they have not been validated in Asian and Indian patients (Table 2).

### Differences in breast cancer between Asian (including indian) versus western women

Aligning with the data published from multiple cohorts, 76% of the panelists agreed that differences exist in breast cancer between Asian and Western women and therefore would require different treatment approaches [46]. Asian women are diagnosed more at a premenopausal age, with larger luminal B tumors, with node-positive disease, and have active tumor micro-environments with frequent *TP53* mutations vs. Caucasian women warranting aggressive treatment strategies [47–50]. The consensus was that Asian (including Indian) younger women have the more aggressive disease (84%); high expression of proliferative genes (80%); and higher involvement of genes involving endocrine resistance (80%) (Table 1). In a study involving a large SEER database of 86,030 patients who underwent Oncotype DX testing, it was found that a large number of Black women were likely to have RS greater than 25 (high-risk requiring chemotherapy) compared to White women with lower accuracy of Oncotype DX in identifying low-risk patients (RS 0–25) [51]. This data along with post-hoc analysis of TAILORx showed differential performance of Oncotype DX in Black vs. White women, a total of 88% agreed that Black women had higher mortality compared to White women of the same type, grade and stage of breast cancer within the same RS score. The consensus guidelines statement is that Asian/Indian patients have a biologically different disease which is more aggressive in younger patients and can have higher expression of poor prognostic genes compared to Western women (Table 2).

### Applicability of an indian made prognostic test, CanAssist breast in making decisions on chemotherapy use

Regarding CanAssist Breast as a prognostic and predictive test, 84% confirmed that it predicted risk of recurrence based on tumor biology; 80% said it is affordable in LMIC; and 76% stated that it predicts risk of recurrence across diverse ethnic backgrounds and geographies (Table 1). CanAssist Breast is the only test that has been extensively validated on Southeast breast cancer patients [36–38]. Along with this data, CanAssist Breast has validation data on Caucasian patients, who are different from its development cohort (Indian). This data clearly demonstrated its unparalleled performance in the cohorts from Europe and USA [41]. Despite the differences in the disease (breast cancer) between Asian and Caucasian women, CanAssist Breast’s prognostication was similar across these diverse cohorts unlike Oncotype DX, MammaPrint. Post-hoc analysis of the TAILORx trial showed altered performance of Oncotype DX with a higher hazard ratio in Black women vs. White women and non-Hispanic women vs. Hispanic women for the same RS category [45]. Likewise, MammaPrint showed lower low-risk proportions in Asian patients compared to European breast cancer patients [52]. Along with this outstanding performance across the various cohorts, CanAssist Breast showed greater than 83% concordance in the low-risk category with Oncotype DX and MammaPrint, the greatest agreement shown between two prognostic tests ever [41, 53]. Optima prelim trial that assessed the agreement between Oncotype DX, MammaPrint, Prosigna, IHC4 and IHC4 Aqua reported a disagreement between these tests in 60.6% of tumors [54]. The accuracy of the test and its cost are the major determining factors in the choice of a prognostic test. With the five times higher price of Oncotype DX and MammaPrint [55, 56] than that of CanAssist Breast [40], the use of these Western multi-gene tests for many patients from India, SAARC countries would be a far-fetched option. The consensus guidelines statement is that CanAssist Breast is a prognostic test that predicts the risk of recurrence; is applicable across ethnic backgrounds and geographies and is affordable in LMIC (Table 2).

### Extended hormonal therapy in high-risk HR + ve, HER2/neu-ve EBC

The use of hormonal therapy for more than 5 years in postmenopausal women reduced the risk of recurrence as well as the risk of contralateral breast cancer was the opinion of 92% of panelists (Table 1). These opinions were based on the results of a placebo-controlled clinical trial and ATLAS trial [57, 58]. In line with the results from aTTOM trial [59] where BCI (Breast Cancer Index) high-risk patients derived significant benefit from extended hormonal therapy, 80% of panelists voted for the opinion that recurrence after 5 years of adjuvant endocrine therapy is seen in patients who have been identified as having high risk based on multi-marker prognostic testing. Another factor worth considering for treating post-menopausal women with extended hormonal therapy is bone health. The results of bone mineral density test will be a guiding factor for prescribing hormonal therapy beyond 5 years. All members of the expert committee agreed that hormonal therapy should be used for male patients (Table 1) and 96% stated the preferred choice of treatment is tamoxifen. The consensus guidelines statement is that hormonal therapy with tamoxifen should be used in male patients; use of hormonal therapy for more than 5 years reduces the risk of recurrence as well as contralateral breast cancer; recurrence after 5 years of endocrine therapy occurs in patients who have been identified as having high risk by multi-marker prognostic testing (Table 2).

### Use of prognostic tests for luminal sub-type

Although there were no questions asked in the survey regarding luminal sub-types, in the final in-person round the panelists discussed the adjuvant systemic treatment strategies for luminal-like patients (Table 4). The panelists concluded that luminal-A like patients have a good prognosis with endocrine therapy alone. Few patients are identified as ‘high-risk’ by the multi-marker prognostic tests and patients with high tumor burden with large tumors or high nodal involvement or poorly differentiated tumors derive benefit from chemotherapy. Patients whose tumors are Luminal-A like are generally suitable for endocrine therapy alone. In case they have high-risk features (on biomarker testing or high tumor burden), chemotherapy should be added. St Gallen’s expert panel also recommends chemotherapy for luminal-A patients with large tumor volume [60]. For patients who have Luminal-B like tumors and are HER2/neu negative, both chemotherapy and endocrine therapy are to be used. If biomarker testing indicates a low risk for cancer recurrence, chemotherapy can be avoided in such patients. Oncotype DX and CanAssist Breast are known to identify high-risk patients from luminal-A like patients and low-risk patients from luminal-B like patients [40, 61].

**Table 4:** Systemic therapy for HR + ve HER2/neu -ve Early Breast Cancer

Sr No	Breast cancer subtype	Systemic therapy recommendations	Comments
1	Luminal A like	Endocrine therapy alone	Chemotherapy to be added if high risk on multi-marker testing and/or high tumor burden (T3/T4 or ≥ 4 LN or G3 tumors involved)
2	Luminal B like, who are HER2/neu –ve	Chemotherapy followed by endocrine therapy	Chemotherapy to be avoided if low risk on multi-marker testing

## Conclusions

**Fig. 1:**
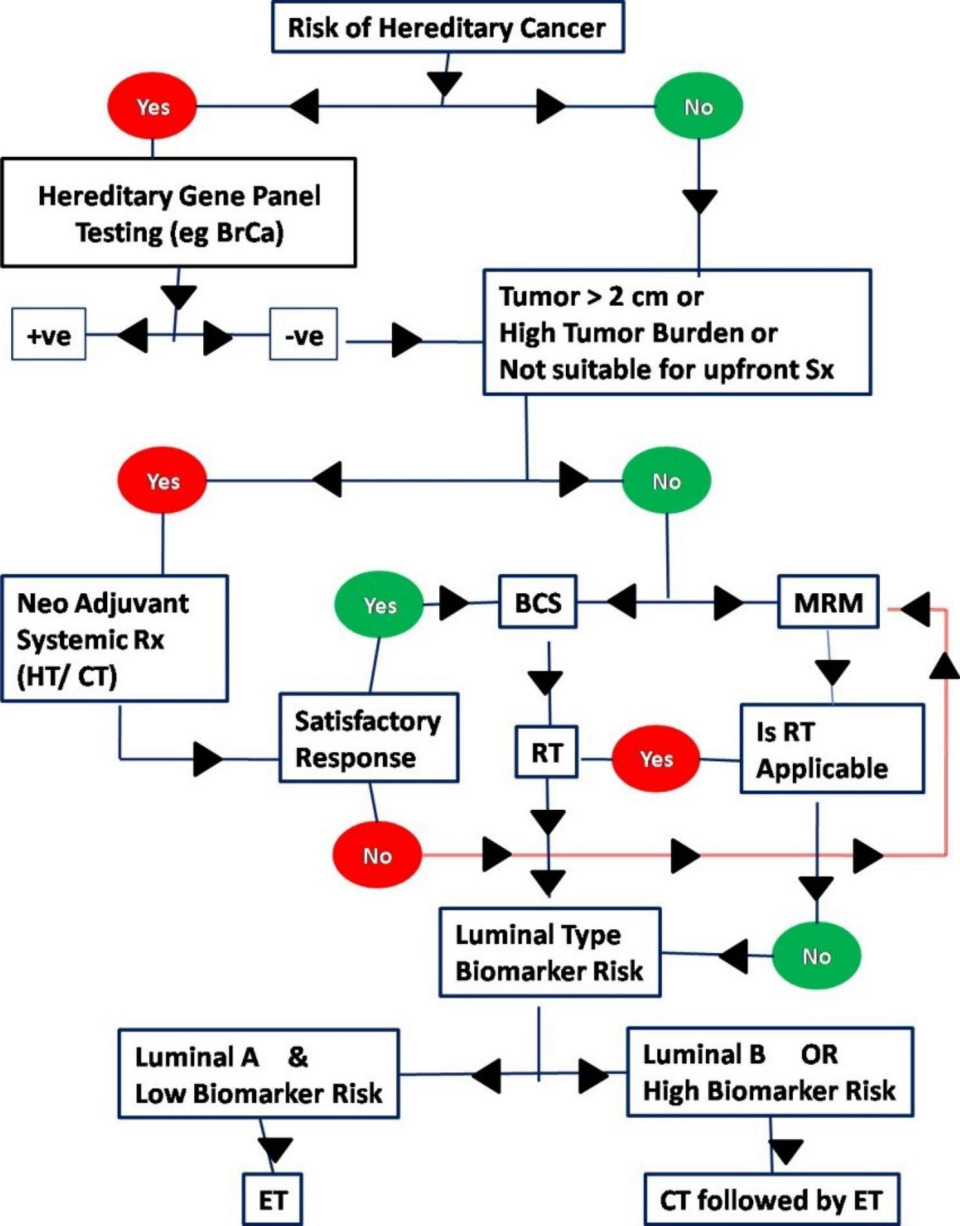
Management decision flowchart for HR+, HER2/neu negative early Breast Cancer

Based on the opinions collected in this survey, the expert committee developed a flow chart (Fig. 1) that the community oncologist can refer to for quick implementation of these consensus guidelines and recommendations; for robust decision making while dealing with patients with HR+/HER2- EBC; and as a handy tool for patient counseling as well as teaching purposes, especially for fellows and postgraduates in oncology.

## Data Availability

The data generated in the current work will be available from the corresponding author on reasonable request.

## Declarations

### Experimental methods

The study does not involve any experimental protocols on human subjects. It uses a method that is well-adapted internationally for conducting a survey of this kind.
